# Hybrid Approach to State Estimation for Bioprocess Control

**DOI:** 10.3390/bioengineering4010021

**Published:** 2017-03-08

**Authors:** Rimvydas Simutis, Andreas Lübbert

**Affiliations:** 1 Department of Automation, Kaunas University of Technology, Kaunas 44249, Lithuania; Rimvydas.simutis@ktu.lt; 2 Department of Biochemie/Biotechnologie, Martin-Luther-Universität Halle-Wittenberg, 06108 Halle, Germany

**Keywords:** State estimation, hybrid modeling, Unscented Kalman Filter, recombinant protein production

## Abstract

An improved state estimation technique for bioprocess control applications is proposed where a hybrid version of the Unscented Kalman Filter (UKF) is employed. The underlying dynamic system model is formulated as a conventional system of ordinary differential equations based on the mass balances of the state variables biomass, substrate, and product, while the observation model, describing the less established relationship between the state variables and the measurement quantities, is formulated in a data driven way. The latter is formulated by means of a support vector regression (SVR) model. The UKF is applied to a recombinant therapeutic protein production process using *Escherichia coli* bacteria. Additionally, the state vector was extended by the specific biomass growth rate µ in order to allow for the estimation of this key variable which is crucial for the implementation of innovative control algorithms in recombinant therapeutic protein production processes. The state estimates depict a sufficiently low noise level which goes perfectly with different advanced bioprocess control applications.

## 1. Introduction

Producers of recombinant therapeutic proteins are increasingly forced to enhance the batch-to-batch reproducibility of their cultivation runs at a high level of productivity. This is not only required to simplify the downstream processing, but it also increases the productivity. Thus, the process must be kept tightly on its predefined optimized track. The important tools for guaranteeing the quality of the cultivation processes are advanced monitoring and feedback control systems. Various bioprocess monitoring and control techniques are described in literature [1,2,3]. They all suffer from the flaw that the online values of the important controlled process variables (biomass, substrate, and product concentrations) are difficult to measure or to estimate to a sufficient accuracy online, and in the current papers on bioprocess control systems the determination of reliable data from the process is usually insufficiently considered.

Here we follow the current development in many engineering subjects, for example, navigation (e.g., [4]), economics (e.g., [5]), tracking moving objects, and process state estimation (e.g., [5]), to increase the estimation accuracy by means of model supported estimation algorithms which combine the a priori knowledge about the process under consideration and the actual measurement data from the online measurement devices [6,7,8].

The most often employed techniques for state monitoring and estimation are based on Kalman Filters, which are also used in modern bioprocess engineering. The original Kalman Filter algorithm provides optimal estimates of measured and unmeasured bioprocess state variables by combining information of linear mathematical models and online measurements [1,8,9].

Importantly, Kalman Filters do not describe the process’ state simply by an *N*-dimensional vector. Instead, the state is considered a set of *N* random variables which, at a given time instant, are described by means of probability distribution density functions. The propagation of these density functions with time step is computed using the dynamical process model. As the original Kalman Filter [10] assumes the model to be linear, the propagation of the density function, such as an *N*-dimensional Gaussian distribution, remains a Gaussian distribution at all times. Such linear propagations do not change the shape of a probability distribution. The new spread of the resulting Gaussian is determined by a new covariance matrix. In nonlinear processes this does not hold. Nonlinear propagations usually change the form of a Gaussian distribution and result in another distribution with at least a skew.

Since most real bioprocess systems are nonlinear, and the state variables are strongly coupled with each other, various extensions of the standard Kalman Filter procedure were proposed. In processes with not too strong nonlinearities, the time increments can be kept so small that the model can be linearly approximated at each time step using a first-order Taylor series linearization of the nonlinear model in order to compute the new covariance matrix. This approach is used in extended Kalman Filters (EKFs), where the density function can be propagated as in the original Kalman Filter. This means that one uses a linear model approximation in order to keep a Gaussian distribution density Gaussian.

In Unscented Kalman Filters (UKFs) one takes another way [11,12,13]. Here the original nonlinear models are directly used and not changed in any way. As these do not conserve the functional form of the density functions upon a mapping to the next time instant, the resulting distribution densities are corrected. As long as the multidimensional density functions are dominated by their means and their covariances, they can be characterized by a small number of points. A simple one-dimensional Gaussian, for instance, is fully determined by its mean µ and its spread (i.e., its mean µ and its inflection points on both sides of the mean at µ ± σ, where σ is the standard deviation). Analogously, in the Unscented Kalman Filter with N state variables, one assumes the distribution density function to be a Gaussian and takes 2*N* + 1 points, the so-called sigma-points, to characterize its form. In order to move the density function from time step to time step, one moves theses characteristic points separately in the state space using the dynamic model. Then, from the transferred points one then determines the mean and the corresponding variances/covariances of the new Gaussian bell. Hence, it is assumed that the new Gaussian distribution density function can be described all the way by means and covariances.

The decisive advantage of the Unscented Kalman Filter is that the dynamic process model and the model that relates the state variables to the quantities that are measured online can be used in their original forms. Only the description of the uncertainty of the states is approximated. The computational complexity of the UKF is similar to that of the Extended Kalman Filter (EKF), and can thus be implemented into the commonly used automation systems.

Although further developments of Kalman Filters also allow for the removal of the restriction on the description of the random variables to simple density functions (an example is the particle filter), they currently cannot be recommended for state estimation in biotechnology. Their relatively small improvements in the estimation accuracy do not justify the much higher computational expenditure they require. Hence, we will consider the Unscented Kalman Filter and propose a hybrid combination of a conventional system of ordinary differential equations to compute the propagation of the state from time step to time step and a data driven model in the form of a Support Vector Machine (SVM) for the mapping of the predicted state variable to the measurement quantities.

## 2. Experimental Data

The data taken here to demonstrate the procedure were taken from Schaepe et al. [14]. There, an *Escherichia coli* strain (BL21:DE3 pLysS) was used which expressed the green fluorescent protein sfGFP [15] under control of the T7-promoter upon induction with IPTG. This protein becomes active within *E. coli*’s cytoplasm and can be detected within the cells with a spectro-fluorimeter.

Importantly, the specific product formation rate π increases monotonically with the cell’s specific growth rate µ. The cultivations were performed in a fed-batch mode at a temperature of 30 °C and pH 7.0 in a stirred tank bioreactor with 15 L maximal working volume.

From all data produced and reported in Schaepe et al. [14], we took the records of the three validation experiments S836 to S838 to demonstrate the process supervision with the proposed hybrid version of the UKF. The corresponding feed rate profiles were determined during tracking experiments, which responded to the changing oxygen uptake capabilities of the cells.

During the cultivations, the UKF only uses the online measured offgas data signals, particularly the cumulative oxygen uptake and carbon dioxide formation rates signals, cOUR and cCPR, respectively, to estimate the biomass and product concentrations as well as the specific biomass growth rate.

These data demonstrate that the estimates which only use online measured data from the offgas analysis very closely predict the biomass and product concentration data, which as offline measured data became available much later only.

## 3. Process Modeling

Kalman Filters [10] require two models; the first is used to move the elements of the state vector within the state space from one time step *t_k−1_* to the next one *t_k_*. The second is the observation model that relates the state vector at time *t_k_* to the actual measurement quantities at that time. The Kalman Filter algorithm estimates the current state of process variables, along with their uncertainties.

### 3.1. State Propagation Model

For the state propagation model, a basic ordinary differential equation system describing the propagation of the initial state with time is used. The conventionally used equation system involves the mass balances around the reactor for the state variables. As such, we consider the biomass, the substrate, and the product. As the process was operated in the fed-batch mode, an additional equation is required that takes into account the change of the working volume *W* with time.

(1) $$\frac{\partial c}{\partial t}=R+\;\frac{F}{W}\left(c_{F}-c\right)$$

(2) $$\frac{dµ}{dt}=0$$

(3) $$\frac{dW}{dt}=F$$

Here, *c* = [X; S; P] is the state vector with the concentrations, X of biomass, S of the substrate, and P of the product. The specific growth rate µ is taken as an additional state variable which can be estimated during the state estimation procedure. It is assumed to be practically constant and only changed by some modeling noise given by the corresponding element of diagonal covariance matrix *V_mod_*. *F* is the substrate feed rate, and *c_F_* is the concentration of the solution fed to the culture. The substrate concentration is the only nonzero element in *c_F_*. It was 600 g/L in this concrete case.

The biochemical conversion is described by the volumetric conversion rate, *R*, which contains the specific conversion rates of the biomass, µ, the substrate, σ, and the product, π. Usually these are modeled by simple or slightly extended Monod expressions. Concretely, the following volumetric conversion rates were taken.

(4) $$R=\left[\left\{µ;-\left(\frac{µ}{Y_{xs}}+\frac{\pi }{Y_{ps}}+m_{s}\right);\;Y_{px}µ\right\}*X\;\right]$$

As the specific biomass growth rate µ was taken as a state variable, its value is taken from the current state estimate of the Unscented Kalman Filter.

**Figure 1:**
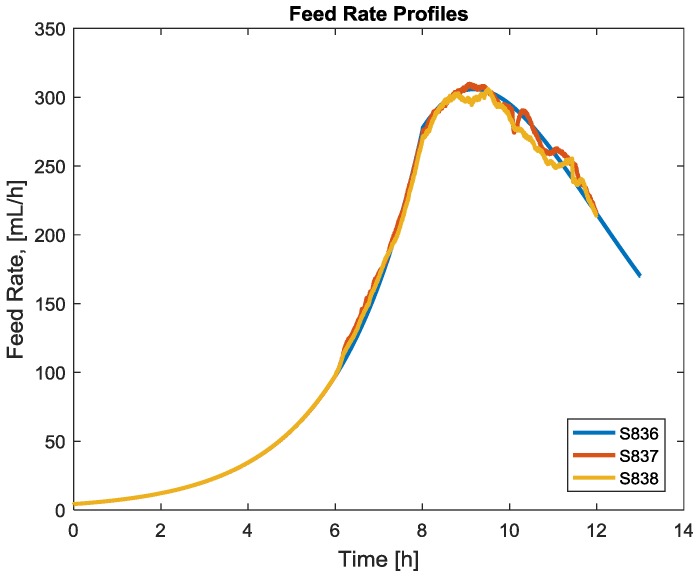
Feed rate profiles from the cultivation experiments used here as the example.

With the initial conditions *c*_0_ for *c*, µ_0_ for the specific biomass growth rate µ, and *W*_0_ for *W*, as well as the feed rate profile *F*(*t*) and the concentrations *c_F_* in the feed, the equation can be solved. The feed rate profiles are manipulated variables and could be measured online (Figure 1).

With the feed rate data depicted in Figure 1, the model can be fitted to process data in order to obtain the free parameters of the dynamic process model, the yields *Y_xs_*, *Y_ps_*, *Y_px_*, and the maintenance coefficient *m_s_*.

### 3.2. Observation Model

The state vector *c* at time *t_k_* corresponds to a number of quantities that can be measured during the cultivation process. The obvious first question, which of the possible measurement variables reflect the most information about the process’ dynamics, can quite easily be answered. For that purpose, it is straightforward to look at the well-established gross reaction equation that describes the biochemical conversion process. It contains, elementwise, the conversion of the significantly changing components with respect to the elements carbon (C), hydrogen (H), oxygen (O), and nitrogen (N). A typical equation is:

C_6_H_12_O_6_ + a O_2_ + b NH_3_ = c CH_1.8_O_0.5_N_0.2_ + d CO_2_ + e H_2_O (5)

It is referred to as the stoichiometric equation of the conversion process, where the coefficients a, b, c, d, and e are stoichiometric coefficients or yields.

As the equation considers only those species, the amounts of which are significantly changing during the biochemical conversion process, this equation gives a direct indication of the quantities that should be measured during the process.

Here we are led to the oxygen consumption (O_2_), the base consumption (NH_3_), and the carbon dioxide (CO_2_) formation. The water formation cannot be considered, as its amount is negligible as compared to the water that is part of the cultivation medium. The water production rate cannot be measured accurately enough and is not considered here.

In a practical application, the corresponding rates, the oxygen uptake rate (OUR), the carbon dioxide production rate (CPR), and the base consumption rate (BCR), are usually measured online during the cultivation process. However, in order to reduce the noise level of the measurement signals, it is advisable to replace the original rate signals by their corresponding cumulative rate signals (e.g., the cumulative oxygen uptake rate cOUR). This does not only reduce the noise level, but it additionally plays to the fact that the important state variables, the biomass and the product concentrations, are cumulative quantities as well.

Hence, we are looking for models that describe the cumulative rates cOUR and cCPR as functions of the state variables *c*. As we usually have measurement sampling rates in the order of 1 Hz of these quantities, while the time constant of the changes in the state variables is in the order of 1 h, the cumulation does not influence the measurement information significantly. These signals follow changes in the biochemical kinetics quickly enough, a fact which was already shown in many closed loop control investigations (e.g., [16]).

The classical textbook relationships between the oxygen uptake rates and the biomass concentrations, such as variants of the Luedeking/Piret equation [17], are not accurate enough as an observation model. Hence, it is straightforward to use data driven models for this purpose, in the sense of learning from the experience with measurement data, where mechanistic models are not yet available to a comparable level of accuracy. Various forms of nonlinear regression models (polynomials, feed forward neural networks, etc.) can be used for modelling these relationships [18].

From the many possibilities, we chose the support vector machine approach [19,20], a regression technique that is an advanced kernel approach. Support vector regression (SVR) techniques require less time and expertise than the artificial neural networks to train the model. This is mainly because SVRs are trained with a structured algorithm (quadratic optimization), which has one unique solution, and it consistently produces the same results when trained with identical data and parameters. Data from new cultivation examples can easily be used to extend and improve an existing SVR model without additional tuning of the model parameters. SVR techniques are also more robust for models with multidimensional inputs.

In our Kalman Filter we need a representation of the measurement quantities cOUR and cCPR as a function of the state variables. We took these data from the recombinant protein cultivation experiments [14] and used general radial basis functions or Gaussian bells as kernels.

**Figure 2:**
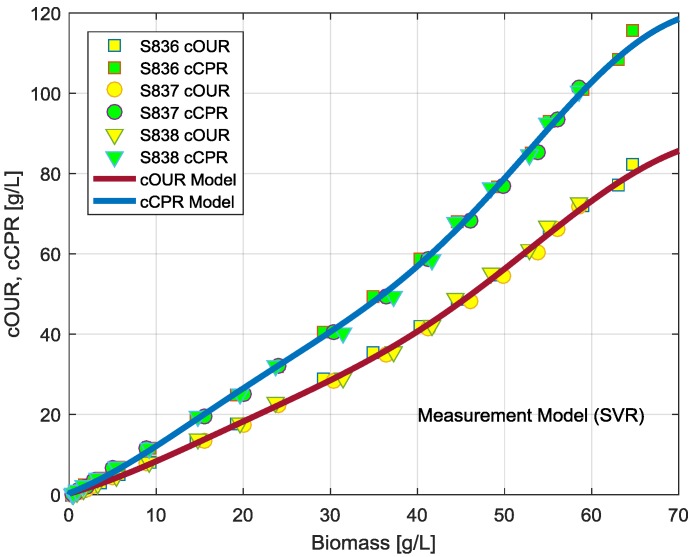
Cumulative oxygen uptake and carbon dioxide production rates signals as a function of the biomass concentration *X*. The curves show a direct evaluation of the support vector regression (SVR) model trained on the data of the cultivations S836, S837, and S838 [14] using the cross validation techniques. cOUR, cumulative oxygen uptake rate; cCPR, cumulative carbon dioxide production rate.

The observation model describing the cumulative oxygen uptake rate and the cumulative carbon dioxide production rate as nonlinear functions of the biomass concentration is presented as lines in Figure 2. The data points (symbols in Figure 2) were taken from the offline measured biomass concentrations and the cumulative OUR and CPR data measured at the corresponding time instants. As can be seen in Figure 2, all records from the three experiments were used to train the SVM model. A cross validation technique was employed using 70% of the data points for the training and 30% for a validation.

## 4. Employing the Unscented Kalman Filter

As all Kalman Filters, the Unscented Kalman Filter UKF is a recursive algorithm that determines the estimate *c*(*t_k_*) at time *t_k_* from the previous estimate *c*(*t_k_*_−1_) [11,12].

It first proposes a state vector *c***^**(*t_k_*) from the previous estimate *c*(*t*_*k*−1_) using the nonlinear process model Ψ, (in our concrete application, the model is described by Equation (1), where the actual state vector *c*(*t*) is [X; S; P; µ]) and computes the corresponding measurement quantities *y*(*t_k_*) from *c***^**(*t_k_*) using the nonlinear observation model H (in our case, this model is presented by support vector regression equations for cOUR and cCPR). The proposal *c***^**(*t_k_*) is then corrected to compute the new estimate *c*(*t_k_*) using the difference between the actually measured values *y*^(*m*)^(*t_k_*) and the computed values *y*(*t_k_*):

*c*(*t_k_*) = *c***^**(*t_k_*) + K (*y*^(*m*)^(*t_k_*) − *y*(*t_k_*)) (6)

where the matrix K that rules the correction of the proposal *c***^**(*t_k_*) depends on the uncertainties of the observations and the transfer model [12]. In this application, the covariance matrixes were taken as diagonal matrices. The initial state covariance matrix had the diagonal elements *V_state_* = diag([.2, .2, .2, 2.0]). The measurement noise *V_meas_*, and *V_mod_*, the model noise, are also incorporated with their diagonal elements Vmeas = [0.01, 0.01], and *V_mod_* = 0.01*V_state_.

**Figure 3:**
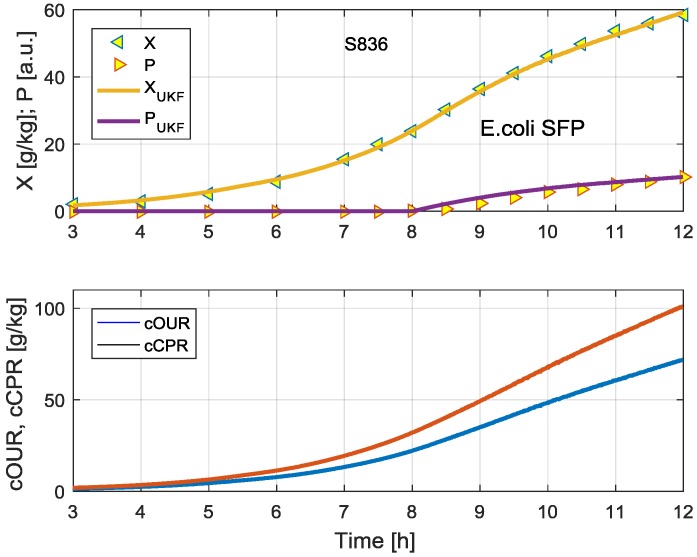
Typical result for the study S836: In the upper plot, the biomass and the product concentration data are displayed as symbols together with the Unscented Kalman Filter (UKF) estimates (lines). In the lower plot the measurement data used in the estimates are depicted.

Figure 3 shows an example of an UKF state estimation of the biomass and the product concentration from measurement data of the cumulative oxygen uptake rate cOUR and the cumulative carbon dioxide production rate cCPR signals based on data (Cultivations S836, S837, and S838) from Schaepe et al. [14]. The symbols shown in Figure 3 are measurement data that were measured offline. They were not used during the estimate of the state variables, and are only taken to show that the estimates are accurate.

The Unscented Kalman Filter software encodes the algorithm described by Wan and van der Merwe [12] (Algorithm 3.1 in that work) and was encoded in Matlab [21]. Therein the SVR regression software was used to train and evaluate the observation model. For that purpose, the generally accessible LIBSVM-software of Chang and Lin [22] was utilized and radial bases functions were used as kernel functions.

**Figure 4:**
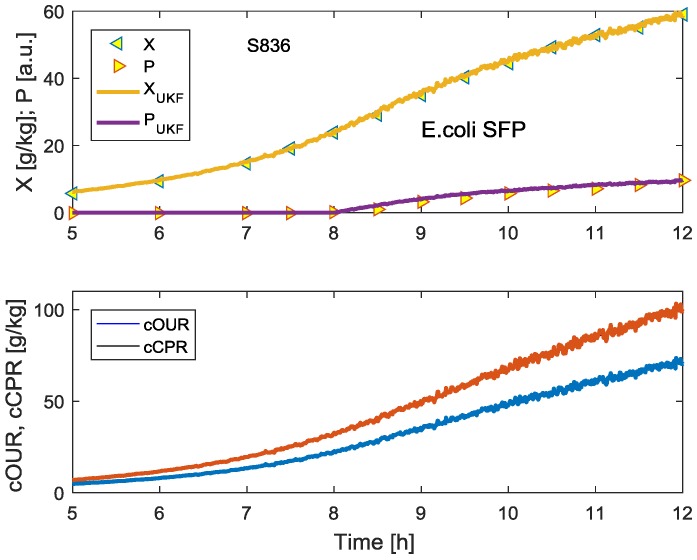
Results corresponding to the graphs in Figure 3 with 2.5% noise on the cOUR and cCPR measurements.

Even if the measurement values cOUR and cCPR are artificially distorted by random noise, for example, by 2.5% of the measured values, the Unscented Kalman Filter does not show much different results in the state variables biomass X and product P concentrations, as shown in Figure 4.

**Figure 5:**
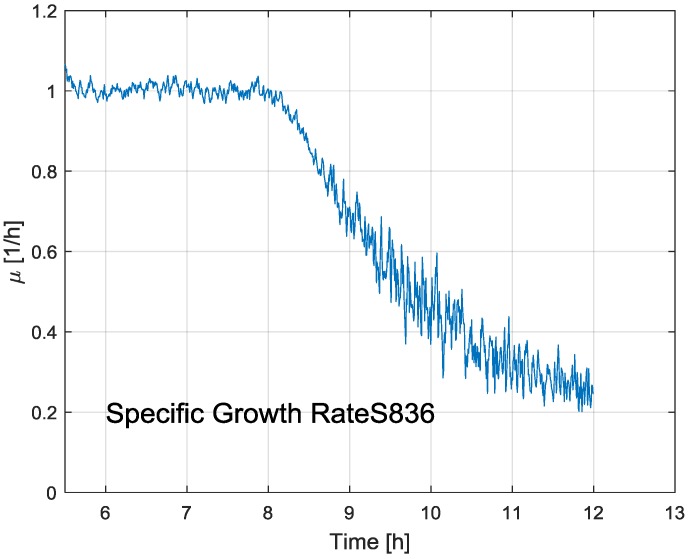
Estimation of the specific growth rate during the cultivation run S836.

The results for the other two cultivation data records are qualitatively the same, and are thus not repeated here. As already stated above, the UKF algorithm was also used for estimating the specific growth rate of the biomass. Figure 5 presents the typical estimation result of the specific growth rate profile across the cultivation.

These estimated online values of biomass, product concentrations, and specific growth rate estimates can then be used in various inferential data analysis and specific growth rate control schemes, as well as for process optimization tasks.

## 5. Conclusions

Process supervision is recommended with Unscented Kalman Filters where the dynamic equations are based on mass balances for the biomass, the substrate, and the product, and formulated by well-established ordinary differential equation systems. As the biomass growth kinetics is not a priori known on the same level of accuracy the specific biomass growth rate µ was taken as an unknown, which is estimated in the same way as the other state variables. The less well-known relationships between the state variables biomass, substrate, and product concentrations and the measurement quantities can be modelled to a sufficient degree of accuracy with modern data-driven methods developed in the machine learning community. The support vector machine technique [19] is one example, advanced neural networks and relevance vector machines [23,24] are other alternatives.

The decisive advantage of this type of nonlinear Kalman Filters is that the process and measurement models can be used directly in the estimation algorithms without any change and without the necessity of linearizing the models. The results show that the hybrid UKF method using a support vector regression model as the observation model delivers satisfactory estimates of the state variables, particularly the most important ones, the biomass and the product concentrations, and even the specific biomass growth rate. The example uses real process data in order to show that the estimation technique is not merely a play with software concepts, but leads to process data that are more accurate and reliable than the separate simulated and measured data.

These accurate estimates of the state variables are well suited for advanced process monitoring and control tasks.
